# Mitochondrial Quantity and Quality in Age-Related Sarcopenia

**DOI:** 10.3390/ijms25042052

**Published:** 2024-02-08

**Authors:** Emanuele Marzetti, Riccardo Calvani, Hélio José Coelho-Júnior, Francesco Landi, Anna Picca

**Affiliations:** 1 Fondazione Policlinico Universitario “Agostino Gemelli” IRCCS, L.go A. Gemelli 8, 00168 Rome, Italy; riccardo.calvani@unicatt.it (R.C.); francesco.landi@unicatt.it (F.L.); 2 Department of Geriatrics, Orthopedics and Rheumatology, Università Cattolica del Sacro Cuore, L.go F. Vito 1, 00618 Rome, Italy; coelhojunior@hotmail.com.br; 3 Department of Medicine and Surgery, LUM University, SS100 km 18, 70010 Casamassima, Italy

**Keywords:** DAMPs, extracellular vesicles, inflammaging, metabolism, mitochondrial biogenesis, mitochondrial DNA, mitochondrial transplantation, mitophagy, muscle aging, muscle plasticity

## Abstract

Sarcopenia, the age-associated decline in skeletal muscle mass and strength, is a condition with a complex pathophysiology. Among the factors underlying the development of sarcopenia are the progressive demise of motor neurons, the transition from fast to slow myosin isoform (type II to type I fiber switch), and the decrease in satellite cell number and function. Mitochondrial dysfunction has been indicated as a key contributor to skeletal myocyte decline and loss of physical performance with aging. Several systems have been implicated in the regulation of muscle plasticity and trophism such as the fine-tuned and complex regulation between the stimulator of protein synthesis, mechanistic target of rapamycin (mTOR), and the inhibitor of mTOR, AMP-activated protein kinase (AMPK), that promotes muscle catabolism. Here, we provide an overview of the molecular mechanisms linking mitochondrial signaling and quality with muscle homeostasis and performance and discuss the main pathways elicited by their imbalance during age-related muscle wasting. We also discuss lifestyle interventions (i.e., physical exercise and nutrition) that may be exploited to preserve mitochondrial function in the aged muscle. Finally, we illustrate the emerging possibility of rescuing muscle tissue homeostasis through mitochondrial transplantation.

## 1. Introduction

Skeletal muscle accounts for about 40% of total body mass in mammals and is essential for balance, posture, and locomotion [1]. This organ also holds remarkable metabolic properties and adaptability to metabolic needs [2] with a role as an endocrine signaling system [3]. Alterations in muscle metabolic plasticity and signaling have been described in the setting of conditions associated with age (e.g., sarcopenia) and muscle atrophy (e.g., disuse).

Sarcopenia is characterized by a reduction in the size and number of myofibers, which preferentially affect fast-twitch type II fibers. This is mostly driven by loss of motor neurons, fast to slow myosin isoform transition (type II to type I fiber switch), and decrease in satellite cell numbers and function [4,5,6]. However, subcellular factors also play relevant roles.

Mitochondria occupy from 2 to 7% of the muscle cell’s volume [7,8]. These organelles are instrumental in supplying ATP for contraction but also participate in the coordination of several cellular processes and activities [9]. These include, among others, redox homeostasis, calcium and iron buffering, amino acid and lipid metabolism, thermogenesis, cellular senescence, and myonuclear apoptosis [10,11]. High-quality functional mitochondria are therefore needed to maintain muscle quality and performance. Mitochondrial quality control is, at least partly, guaranteed by the coordinated activity of mitochondrial biogenesis and mitophagy that removes bioenergetically impaired and low-quality mitochondria [12].

Additional systems have been implicated in the regulation of muscle plasticity and trophism such as the tightly modulated and complex interplay between the stimulator of protein synthesis, mechanistic target of rapamycin (mTOR), and the inhibitor of mTOR, AMP-activated protein kinase (AMPK), that promotes muscle catabolism [13].

Herein, we provide an overview of the evidence linking mitochondrial function and quality with muscle homeostasis and performance and discuss the main pathways elicited by their imbalance during age-related muscle wasting and associated conditions.

## 2. Age-Related Changes in Muscle and Mitochondrial Function

### 2.1. Age-Related Changes in Muscle Mass, Strength, and Function

Sarcopenia is associated with several negative health-related outcomes [14,15] and impacts the prognosis of chronic conditions, including cancer [16,17], diabetes [18], and heart failure [19]. A greater risk of all-cause mortality has also been reported in older adults with sarcopenia compared with non-sarcopenic peers [20,21,22].

Skeletal muscle mass remains almost steady until the age of 60 [23] when it starts to decline at a rate of 4.7% and 3.7% per decade in men and women, respectively, past 70 years of age [24]. Rates of muscle mass loss differ between the upper and lower limbs, with the former exhibiting decreases of less than half of the lower limbs [1]. Furthermore, sex-specific declines have been described, with men having greater age-related losses of muscle mass than women [25].

Muscle strength is the maximal force produced by a muscle or group of muscles under predetermined conditions. Muscle strength starts to decline earlier, approximately during the fourth decade of life, and proceeds at steeper rates (12–15% per decade) past the age of 50 with a peak between 65 and 80 years of age [26,27,28]. Age-related changes in strength also show muscle-specificity [29,30,31] and are often accompanied by an even more rapid decline in muscle power (i.e., the ability to generate strength rapidly [31]) [32]. These variations are multifactorial involving both the nervous and musculoskeletal system [33,34].

Lower limb muscle power has repeatedly been indicated as a better predictor of mobility disability in older adults than muscle strength [35,36,37,38]. Significant relationships between lower limb muscle power and the performance on several tests (e.g., stair climbing, tandem gait, habitual gait velocity, maximal gait velocity, short physical performance battery) have been identified in adults older than 70 [35]. Other studies have also indicated that handgrip strength is associated with lower limb strength and physical function in old community-dwellers [39,40]. Taken as a whole, these findings indicate that declines in muscle strength and power are major contributors to the development of mobility disability in older adults, with muscle power decreasing more rapidly than muscle strength. Among the many factors contributing to age-related declines in muscle strength and power, muscle mass is of utmost importance.

**Figure 1:**
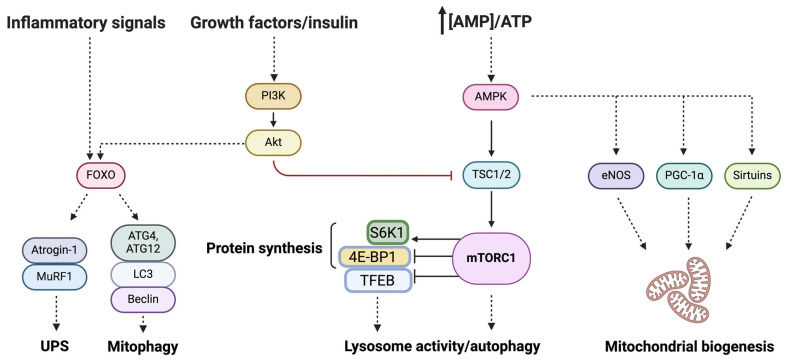
Schematic representation of the main molecular pathways that become dysregulated in age-related sarcopenia. Abbreviations: Akt, protein kinase B; AMPK, AMP-activated protein kinase; 4E-BP1, eIF4E-binding protein 1; eNOS, endothelial nitric oxide synthase; FOXO, Forkhead box protein O; ATG, autophagy-related protein; LC3, microtubule-associated proteins 1A/1B light chain 3A; PI3K, phosphoinositide 3 kinase; S6K, S6 kinase; TFEB, transcription factor EB; TSC, tuberous sclerosis protein; UPS, ubiquitin proteasome system. Created with BioRender.com (accessed on 30 January 2024).

A set of pathophysiological changes encompassing both the nervous system [41,42,43,44,45,46] and muscle ultrastructural and functional disarrangements [47,48] accompany the development of sarcopenia and decline in physical performance. A great deal of research has been devoted to dissecting the molecular pathways underlying these variations (Figure 1) and identifying biomarkers that reflect these intricated processes at the systemic level [49,50,51]. These aspects are discussed in the following sections.

### 2.2. Mitochondria and Muscle Aging

In the setting of an elevated workload, the muscle boosts the rate of substrate utilization for energy production, while it undergoes a moderate increase in energy consumption during periods of prolonged low-intensity contractions [52]. Muscle energy requirements increase by about 100-fold from rest to contraction. The actin–myosin cross-bridge cycling during muscle contraction requires a substantial amount of energy; yet, the availability of promptly accessible ATP is only enough for ~2 s of continuous contraction. Energy provision through the phosphocreatine system ensures ATP supply for about 10 s to sustain a quick burst of activity [53]. Hence, newly synthetized ATP will soon be needed to support muscle contraction. Both anaerobic and aerobic processes are engaged to sustain ATP generation and meet the energy requirements of contracting muscles. A long-term sub-maximal exercise activity is fed by aerobic metabolism; but, beyond a certain threshold of exercise intensity, the contribution of anaerobic metabolism becomes prominent [52]. Therefore, muscles derive most of their energy from mitochondrial oxidative phosphorylation as a primary source.

Mitochondrial content, distribution (subsarcolemmal or intermyofibrillar), and ultrastructure vary significantly across muscles and fiber types. Dense and interconnected mitochondria are typically found in red muscles enriched in slow-twitch type I fibers, while white muscles mostly containing fast-twitch type II fibers have a lower mitochondrial content [54]. Slow-twitch type I muscles show high resistance to fatigue while displaying a lower capacity of generating force due to the smaller size of fibers and motor units [55]. Conversely, fast-twitch type II muscle fibers, classifiable into the two subtypes, IIa (oxidative) and type IIx (glycolytic) with intermediate and low mitochondrial content, respectively, show large cross-sectional areas and motor unit size [55]. Such ultrastructural features confer these fibers a greater force-generating capacity with increased fatiguability due to a lower mitochondrial content and higher reliance on anaerobic metabolism. Thus, structural and metabolic differences of myofibers correspond to specific muscle responses to metabolic stimuli and may also determine the susceptibility to fiber atrophy in coordination with other signaling pathways [54].

Along with the progressive age-related decline in muscle mass, strength, and function, mitochondrial content and respiration have also been shown to decrease with aging in human skeletal muscle [9,56]. A reduced content of mitochondria DNA (mtDNA) and proteins, including those involved in the Kreb’s cycle and components of the electron transport chain (ETC), has been described in aged muscles [57,58,59,60]. A reduced size of intermyofibrillar mitochondria as well as thinner layers of subsarcolemmal organelles have also been reported [61,62,63]. However, proteins belonging to the mitochondrial import machinery and ETC complex assembly do not seem to be significantly affected by age [61,64]. The frequency of mtDNA deletions and point mutations increases with advancing age but reaches a critical level late in life when mitochondrial function and signaling are likely to be already compromised [65,66]. Therefore, alterations in the expression of the protein import machinery as well as ETC constituents and mtDNA integrity may not be the primum movens of mitochondrial dysfunction during aging.

A decrease in both RNA and protein expression of the master regulator of mitochondrial biogenesis, peroxisome proliferator-activated receptor-gamma coactivator 1 alpha (PGC-1α), and downstream signaling targets has been reported in slow- and fast-twitch muscle fibers during aging [62,67]. Protein levels of PGC-1α have been shown to correlate with walking speed in healthy older adults [68], while the overexpression of PGC-1α in mice with muscle loss due to injury preserves mitochondrial content and muscle strength [69]. The age-associated decline in mitochondrial function has also been shown to be counteracted by endurance exercise via increasing PGC-1α expression, an effect that cannot be achieved in mice knocked out for PGC-1α [70]. Therefore, a reduced expression of PGC-1α and the consequent lower transcription of nuclear genes encoding for mitochondrial proteins may be a major factor underpinning the decrease in organelle content during aging.

A role as a modulator of mitochondrial biogenesis has also been attributed to the mitochondrial transcription factor A (TFAM). TFAM expression is under the control of the PGC-1 transcriptional regulators via activation of nuclear respiratory factor 1 and 2 (NRF-1 and NRF-2) and the estrogen related receptor alpha (ERRα) [71,72]. TFAM is a histone-like protein of 25 kDa with two high-mobility group box domains that binds without sequence specificity to mtDNA for wrapping purposes. TFAM is also able to bind to mtDNA regions that serve relevant roles in overall mitochondrial genome homeostasis. For instance, the binding of TFAM to the displacement loop (D-loop) region of mtDNA is implicated in both mtDNA replication and transcription initiation [73,74]. Instead, its binding to mtDNA outside the promoter region enables TFAM activity participate to mtDNA stability and repair [73,74,75]. Variations in TFAM protein expression have been reported with aging in different tissues, including the skeletal muscle, and are prevented by calorie restriction [76], a well-studied anti-aging intervention. Furthermore, changes in TFAM binding to mtDNA have been indicated as a possible mechanism whereby PGC-1α affects mitochondrial content and function during aging [76,77,78,79].

Results from preclinical studies showed accelerated sarcopenia in transgenic mice knocked out for the antioxidant enzyme superoxide dismutase 1 (SOD1). These mice display reduced mitochondrial bioenergetics, rapid induction of mitochondrial-mediated myonuclear apoptosis, and degeneration of neuromuscular junctions [80]. Therefore, an enhancement in mitochondrial biogenesis and function is envisioned as a strategy to counteract the age-related decline in physical function.

Reduced levels of physical activity negatively impact mitochondrial capacity [81], while physical exercise triggers mitochondrial biogenesis via PGC-1α [82]. In a recent study, the effects of physical activity were evaluated in the muscle of young and old individuals with similar habitual physical activity levels [83]. Exercise-trained older adults and physically impaired peers were also compared. As an effect of aging, declines in mitochondrial and muscle function occur regardless of physical activity levels. However, an increase in physical activity can reverse the effects of aging on mitochondrial activity [83]. Also, alterations in iron handling and mtDNA maintenance have been described during aging in the muscles of physically inactive older adults independent of functional status [84,85]. Perturbations in cellular and mitochondrial iron homeostasis in the skeletal muscle of older adults with low physical performance have been related to reduced mitochondrial quality and possibly contribute to a loss of mtDNA stability. These findings indicate that muscle iron metabolism may be a target for interventions against muscle aging.

Sex-associated differences have also been identified in the expression profile of genes of the vastus lateralis muscle of old individuals compared with younger controls [86]. While the core processes associated with skeletal muscle aging were detected in both men and women, their magnitude diverged in a sex-specific manner [86]. This is a relevant observation, as it may indicate that age-related sex-specific events, such as menopause, are not necessarily reflected by variations in signaling pathways in the muscle but are rather the consequence of sex-specific aging trajectories. The most differentially expressed genes in men were those related to oxidative phosphorylation, while in women were those involved in cell proliferation via AKT signaling [86].

mTOR is another major regulator of myocyte homeostasis [13]. Hyperactivation of mTOR during aging has a negative impact on muscle protein synthesis and favors muscle loss [87]. In preclinical models, the administration of rapamycin analogs (rapalogs) and derivatives has been shown to prevent age-related muscle loss [88]. Furthermore, calorie restriction triggers AMPK signaling to promote a set of responses, including autophagy and mitochondrial biogenesis, leading to the preservation of muscle quality during aging [89].

A recent study investigated the relationship between markers of mitochondrial quality (e.g., autophagy, mitophagy, lysosomal degradation), vastus lateralis muscle composition, and measures of physical performance in young and old physically inactive participants [90]. The study showed that old participants had smaller muscle volumes and lower values of muscle tissue composition index (i.e., the ratio between the muscle and intermuscular adipose tissue volume) compared with young controls [90]. Protein expression levels of the autophagy marker p62 and the mitophagy mediator BCL2/adenovirus E1B 19 kDa protein-interacting protein 3 (BNIP3) were higher in old participants. Furthermore, a negative correlation was identified between these mediators and the tissue composition index. A negative relationship was identified between protein levels of p62 and BNIP3 and the performance on the 5-time sit-to-stand test [90]. Taken as a whole, these findings indicate that the muscle tissue composition of the lower extremity, muscle performance, and mitochondrial quality are interrelated parameters. Deciphering such relationship may help unveil new pathways amenable for therapeutic development. These points will be discussed more in detail in the next section.

In addition to the molecular pathways described, a set of soluble mediators produced by the contracting muscle and collectively referred to as myokines have been attributed signaling roles in the skeletal muscle [91]. Myokines (e.g., myostatin, irisin), mediators released by the adipose tissue (e.g., adiponectin, apelin, leptin, growth differentiation factor 15), and others produced by the liver (e.g., fibroblast growth factor 21) have been proposed to contribute to shaping muscle metabolism and structure [91]. However, further research is warranted to confirm whether a dysregulation in these signaling routes plays a role in sarcopenia [92].

## 3. Bridging Energy Production to Organelle Quality: The Role of Mitophagy in Muscle (Patho)physiology

High-quality mitochondria are of the utmost importance to supporting the activities of energy-demanding tissues. Mitochondrial quality is achieved through the balance of at least two opposed but complementary processes: mitochondrial autophagy (mitophagy) and biogenesis.

**Figure 2:**
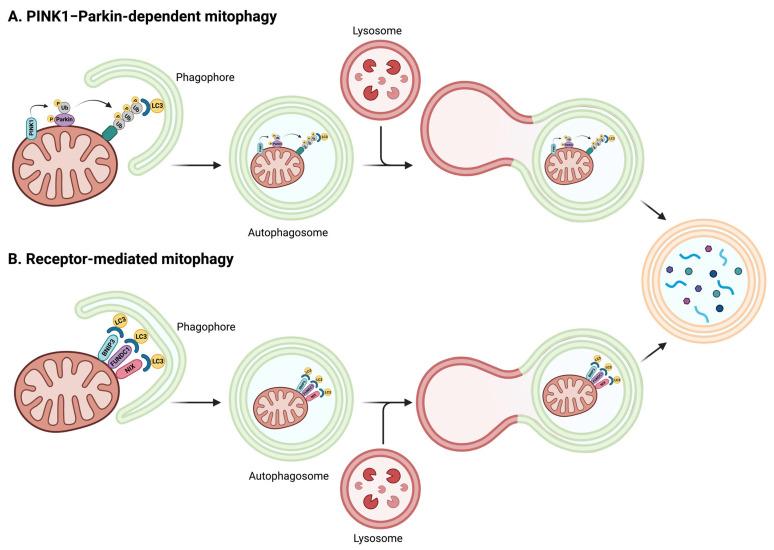
Schematic representation of the events involved in PINK1–Parkin-dependent and PINK1–Parkin-independent mitophagy. (**A**) In the PINK1–Parkin-dependent pathway, ubiquitination of adaptor proteins at the mitochondrial surface is coordinated by PINK1 and Parkin protein activity. In the setting of reduced mitochondrial membrane potential, PINK1 cleavage is abrogated, which leads to PINK1 accumulation at the organelle surface. This is followed by the recruitment and phosphorylation of the E3 ubiquitin ligase Parkin that becomes active and, in turn, ubiquitinates proteins in the mitochondrial outer membrane. PINK1-guided phosphorylation of newly generated ubiquitin moieties also produces phospho-ubiquitin chains exposed to the cytosol that act as docking sites for the recruitment of adaptor proteins and LC3 to guide mitophagosome formation and mitochondrial clearance. (**B**) PINK1–Parkin-independent mitophagy involves BNIP3, NIX, and FUNDC1 mitophagy protein receptors at the mitochondrial surface for the recruitment of LC3 and mitophagosome formation. Abbreviations: BNIP3, BCL2/adenovirus E1B 19 kDa protein-interacting protein 3-like; FUNDC1, FUN14 domain-containing protein 1; LC3, microtubule-associated proteins 1A/1B light chain 3A; NIX, Nip3-like protein X; PINK1, PTEN-induced putative kinase 1; Ub, ubiquitin. Created with BioRender.com (accessed on 18 December 2023).

Intra- and extracellular factors trigger the identification and removal of damaged or unnecessary organelles via mitophagy. This process is enacted via ubiquitination of adaptor proteins at the mitochondrial surface under the coordination of phosphatase and tensin homologue (PTEN)-induced putative kinase 1 (PINK1) and Parkin (i.e., PINK1–Parkin pathway) [93] or the recruitment of mitophagy protein receptors (i.e., PINK1–Parkin-independent mitophagy) at the mitochondrial surface [94,95] (Figure 2).

PINK1 is constitutively cleaved and inactivated at the outer mitochondrial membrane (OMM). In the event of reduced mitochondrial membrane potential, PINK1 cleavage is abrogated and its degradation is inhibited, which leads to PINK1 accumulation at the organelle surface. Herein, PINK1 recruits the E3 ubiquitin ligase Parkin and, via its phosphorylation, unleashes its enzymatic activity with the subsequent ubiquitination of OMM proteins. PINK1-guided phosphorylation of newly generated ubiquitin moieties also produces phospho-ubiquitin (ph-Ub) chains exposed to the cytosol that act as docking sites for the recruitment of adaptor proteins like Optineurin and NDP52 [93]. The relocation of the autophagic adaptor protein p62 at the mitochondrial surface is also essential for organelle clearance. The coordinated activity of adaptor and receptor proteins with the microtubule-associated proteins 1A/1B light chain 3A (LC3) enables the buildup of a double lipidic membrane that encloses and isolates the mitochondrion within a structure called mitophagosome, which thereafter fuses with lysosomes. Here, hydrolases execute mitochondrial degradation and cargo recycling.

The PINK1–Parkin-independent mitophagy involves BCL2/adenovirus E1B 19 kDa protein-interacting protein 3-like (BNIP3), Nip3-like protein X (NIX), and FUN14 domain-containing protein 1 (FUNDC1) for the recruitment of LC3 and mitophagosome formation [94]. PINK1–Parkin-independent mitophagy converges toward the same PINK1–Parkin-guided LC3-dependent mitophagosome formation that in this case operates via BNIP3-mediated stabilization of PINK1- and NIX-modulated Parkin recruitment [96,97].

Age-related impairments in mitochondrial function have been described in the muscles of old individuals in conjunction with reductions in muscle strength and walking performance [98,99,100]. However, in several instances, reduced mitochondrial bioenergetics are not paralleled by lower mitochondrial mass, possibly indicating the persistence of dysfunctional mitochondria owing to the inefficient removal by mitophagy. Because of a perturbed mitophagy, mitochondrial biogenesis could also be stalled [101,102], making it difficult to understand which of the two processes becomes impaired first. Physically frail and sedentary old women show reduced expression of the genes of the mitophagy mediators *PARK2*, *ATG7*, *BECLIN1*, and *BNIP3* in the skeletal muscle [103]. The analysis of muscle biopsies from old physically active men revealed lower protein levels of Parkin compared with young controls [104]. Negative correlations have also been identified between muscle volume and protein levels of p62 and BNIP3 in physically inactive older adults [90]. Finally, high protein levels of ETC complexes have been described in the gastrocnemius muscle of individuals with peripheral artery disease together with impaired mitophagy and reduced mitochondrial function [105,106]. This may be the result of at least two non-mutually exclusive processes. The first involves the accrual of mitophagy receptors at the mitochondrial surface not followed by lysosomal-guided clearance, thus resulting from a stalled mitophagy flux. The second may be due to energy shortage consequent to sustained mitochondrial dysfunction that hampers the energetically expensive mitophagy execution. Additional investigations are needed to clarify this complex regulation.

Finally, as an alternative route to mitophagy, the generation of mitochondrial-derived vesicles (MDVs) has been identified and characterized in older adults with physical frailty and sarcopenia (PF&S). These vesicles have been found to carry components of the mitochondrial ETC [51]. This finding has been related to the metabolic and inflammatory profile as well as mitochondrial dyshomeostasis observed in individuals with PF&S [51]. Indeed, MDVs can be considered damage-associated molecular patterns (DAMPs) generally released from injured cells and that trigger inflammation. This inflammatory response can be elicited via receptors/systems of innate immunity, including the cytosolic cyclic GMP–AMP synthase-stimulator of interferon genes DNA sensing system that is curtailed by efficient mitophagy [107]. Therefore, dysfunctional mitophagy and decline in the mitochondrial quality control processes in skeletal myocytes during aging may ignite mitochondrial damage and the propagation of sterile inflammation via the release of DAMPs. Additional investigation is warranted to confirm this hypothesis for its possible exploitation as a target for interventions against PF&S.

## 4. Mitochondrial Function Recovery: From Energizing Mitochondria to Organelle’s Transplantation

Exercise is a powerful trigger of mitochondrial biogenesis. However, the exercise modality and duration necessary for boosting the mitochondrial biogenesis, quality, and function are still open questions especially in older adults [108].

As per the effects of different types of exercise, endurance training seems to be more effective than resistance exercise to stimulate mitophagy activation and/or execution. Indeed, while an increase in markers of mitochondrial biogenesis either at the transcriptional or translation level has been observed in the muscle or peripheral blood mononuclear cells of older adults engaged in resistance training, no variations were found in mitophagy markers [109,110]. Accordingly, two weeks of resistance training in middle-aged men following lower-limb immobilization was not accompanied by changes in the expression of mitophagy mediators [111]. Conversely, higher levels of Parkin and PINK1 protein have been reported in the muscle of long-term adult runners [112]. Similarly, old cyclists showed increased protein levels of Parkin and LC3 compared with age-matched sedentary controls [113].

The pivotal contribution of mitochondrial function and quality to muscle homeostasis and performance is also epitomized by increasing evidence showing that mitochondrial transplantation may be a therapeutic strategy for the bioenergetic reprogramming of diseased tissues, including the skeletal muscle [114,115,116].

Pioneering studies have investigated the ability of mitochondria transplantation to enhance the recovery from ischemia–reperfusion injury in the setting of myocardial ischemia [117,118]. The injection of autologous respiration-competent mitochondria from non-ischemic heart zones into ischemic regions prior to reperfusion decreased infarct size and increased cell survival in rabbits [119]. These initial findings were supported by subsequent larger investigations [117,120] and eventually reached a clinical stage [121]. Pediatric patients needing extracorporeal membrane oxygenation due to ischemia–reperfusion injury were the first to be enrolled in a trial on autologous mitochondrial transplantation. During surgery, autologous mitochondria purified from skeletal muscle samples were administered intramyocardially [121]. Subsequently, clinical trials have been designed to evaluate the effectiveness of mitochondrial transplantation as a therapeutic strategy in various clinical conditions (NCT02586298, NCT02851758, NCT04998357, NCT04976140). More recent is the finding that transplanting intermyofibrillar mitochondria from murine skeletal myocytes to myoblasts improved myoblast bioenergetics [116]. Human fibroblasts harboring mtDNA mutations were also successfully transplanted with murine muscle mitochondria and showed improved mitochondrial dynamics, metabolism, and reduced levels of reactive oxygen species [116]. The mitochondrial transfer occurred via extracellular vesicles, gap junctions, micropinocytosis, and tunneling nanotubes, thereby paving the way for less invasive techniques for organelle transplantation that exploit vesicle trafficking [116]. Finally, the incorporation of mitochondria into mesenchymal stem cells following organelle transplantation has been shown to improve the repair of arterial, lung, and cardiac tissue [122,123]. Moreover, the systemic delivery of mitochondria after barium chloride-induced muscle injury has been shown to improve muscle regeneration and restore function [124]. Whether this approach may represent a feasible and successful strategy to rescue mitochondrial function in the aged muscle warrants exploration.

## 5. Conclusions

Mitochondrial quality and function are of the utmost importance for achieving optimal skeletal myocyte bioenergetics and have been implicated in preserving muscle quality and performance during aging. On the one hand, a tight coordination between mitochondrial biogenesis and mitophagy ensures a pool of high-quality and fully functional organelles in the muscle. On the other hand, muscle plasticity and trophism rely on the complex regulation of the stimulator of the protein synthesis, mTOR, and the inhibitor of mTOR, AMPK, that promotes muscle catabolism. The maintenance of mitochondrial quality and the stimulation of mitochondrial biogenesis via nutritional and/or physical activity programs have shown success at delaying age-related muscle wasting. The local delivery of functional organelles as an approach to rescue muscle function during aging warrants investigation.

## Data Availability

Not applicable.
